# A systematic review of data mining and machine learning for air pollution epidemiology

**DOI:** 10.1186/s12889-017-4914-3

**Published:** 2017-11-28

**Authors:** Colin Bellinger, Mohomed Shazan Mohomed Jabbar, Osmar Zaïane, Alvaro Osornio-Vargas

**Affiliations:** 1 Department of Computing Science, University of Alberta, Edmonton, Canada; 2 Department of Paediatrics, University of Alberta, Edmonto, Canada

**Keywords:** Epidemiology, Air pollution, Exposure, Data mining, Big data, Machine learning, Association mining

## Abstract

**Background:**

Data measuring airborne pollutants, public health and environmental factors are increasingly being stored and merged. These big datasets offer great potential, but also challenge traditional epidemiological methods. This has motivated the exploration of alternative methods to make predictions, find patterns and extract information. To this end, data mining and machine learning algorithms are increasingly being applied to air pollution epidemiology.

**Methods:**

We conducted a systematic literature review on the application of data mining and machine learning methods in air pollution epidemiology. We carried out our search process in PubMed, the MEDLINE database and Google Scholar. Research articles applying data mining and machine learning methods to air pollution epidemiology were queried and reviewed.

**Results:**

Our search queries resulted in 400 research articles. Our fine-grained analysis employed our inclusion/exclusion criteria to reduce the results to 47 articles, which we separate into three primary areas of interest: 1) source apportionment; 2) forecasting/prediction of air pollution/quality or exposure; and 3) generating hypotheses. Early applications had a preference for artificial neural networks. In more recent work, decision trees, support vector machines, k-means clustering and the APRIORI algorithm have been widely applied. Our survey shows that the majority of the research has been conducted in Europe, China and the USA, and that data mining is becoming an increasingly common tool in environmental health. For potential new directions, we have identified that deep learning and geo-spacial pattern mining are two burgeoning areas of data mining that have good potential for future applications in air pollution epidemiology.

**Conclusions:**

We carried out a systematic review identifying the current trends, challenges and new directions to explore in the application of data mining methods to air pollution epidemiology. This work shows that data mining is increasingly being applied in air pollution epidemiology.

The potential to support air pollution epidemiology continues to grow with advancements in data mining related to temporal and geo-spacial mining, and deep learning. This is further supported by new sensors and storage mediums that enable larger, better quality data. This suggests that many more fruitful applications can be expected in the future.

## Background

The decreasing costs of remote sensors for measuring airborne agents, along with the increasing availability of environmental and clinical data, has led to an explosion in the number of pollution datasets available for analysis. These datasets often have a very large number of samples and tend to have a significant number of variables with mixed degrees of dependencies. These big datasets come with complexity that renders it difficult to rely on traditional epidemiological or environmental health models to analyze them. As a result, new methods of analysis are required in order to advance our understanding of the data. Data mining and machine learning methods from computing science present a wide array of scalable and reliable methods that have performed well on similar problems in other domains. This has inspired a burgeoning field of research within Environmental Health aimed at the adoption of data mining methods to analyze modern, big datasets in air pollution epidemiology inefficient and effective ways.

Data mining is the computational process that is often applied to analyze large datasets, discover patterns, extract actionable knowledge and predict outcomes of future or unknown events. Methods used in this process come from a combination of computational disciplines including Artificial Intelligence, Statistics, Mathematics, Machine Learning, and Database Systems. Apart from the core methods used to carry out the analysis, the process of data mining can involve various preprocessing steps prior to executing the mining algorithm. In addition, a post-processing stage is typically employed to visualize the results of the analysis (i.e. recognized patterns or retrieved information) in an intuitive and easy-to-communicate manner. In this review, we limit our scope to focus on core data analysis techniques as they have been applied to the field of air pollution epidemiology and reported within the air pollution epidemiology literature.

In a broad sense, there are two major paradigms of algorithms: prediction and knowledge discovery. Within these, there are four sub-categories: 1) Classification and regression, 2) Clustering, 3) Association Rule Mining, and 4) Outlier/Anomaly Detection. In addition, there are some relatively new and exciting areas of data analysis, such as spatial data mining and graph data mining, that have been made possible via the building blocks of data mining methods.

According to the best of our knowledge, there are no studies that investigate the depth and breadth of the application of data mining methods within air pollution epidemiology. With this in mind, we perform an investigation to identify which data mining methods have been applied, and to which areas of air pollution epidemiology they have been applied to. Our goal is to point domain researchers to preexisting data mining applications in their areas, and related areas, as well as advance their understanding of the potential of data mining and inspire them to explore further research avenues.

## Methodology and paradigms of data mining algorithms

Data mining algorithms are particularly beneficial on complex datasets with a large number of variables and samples. With respect to knowledge discovery, they add insight into high-dimensional problems where traditional statistical methods often fail. Similarly, machine learning algorithm can induce accurate predictor functions from complex, high-dimensional datasets where statistical and mathematical methods, such as regression, can be prone to inaccuracies and be difficult to apply due to their underlying assumptions.

### Considerations for applying data mining

In order to implement a successful data mining solution, the user must analyze and formalize their objective. The problem objective guides the user to the appropriate paradigm of learning algorithm. If the objective is to identify hidden groups in data or identify associations between key variables in the data, the users are interested in knowledge discovery and will want to select a clustering or association mining algorithm. Alternatively, the objective might be to induce a predictive model that can classify samples as belonging to a particular category, such as poor air quality, or a real-valued outcome, such as the air quality index.

A large and growing number of algorithms belong to the prediction paradigm and the knowledge discovery paradigm. How to choose between the methods within each paradigm is a topic in its own right. To assist practitioners that are new to the application of machine learning algorithms, Domingos discusses the some of the key considerations in [1].

When making this decision, the user should consider the complexity of the problem and the amount of data available. A simple, linear classifier, for example, will be ineffective on a complex non-linear classification problem. A large volume of data will facilitate the use of advanced learning algorithms, such as deep artificial neural networks [2], however, it also forces users to consider questions related to storage, memory and training time.

In general, it is widely understood that there is no silver bullet when it comes to learning algorithms. From an application perspective, a good practice is to select a small, diverse set of algorithms from the paradigm of relevant methods, test them individually and select the one that best meets the performance objectives. Alternatively, grouping a diverse set of models to form an ensemble of predictors has been demonstrated to be an effective solution in theory and practice [3]. Within the surveyed literature, for example, [4] applied an ensemble formed of neural networks, support vector machines, Gaussian processes, decision trees and random forests.

Once a set of potential algorithms has been selected, the models that are induced by each algorithm over the available dataset must be evaluated in order to select the one model, or ensemble, that is most likely to preform the best on the prediction task in the future. This is an area of research that is presented in *Error Estimation for Pattern Recognition* [5] and *Evaluating Learning Algorithms* [6].

The paradigms of learning that have most widely been applied in air pollution epidemiology can be categorized as prediction-based or knowledge discovery methods.

### Value prediction

Value prediction is a common and widely applicable area of data mining in which the objective is to take in a set of variables related to an instance drawn from an underlying sample population and predict the corresponding value. Depending on the nature of the application, the user will choose either a data mining algorithm that makes categorical predictions (a classifier) or numeric predictions (similar to regression.) Typical classification algorithms include decision trees, Bayesian classifiers, support vector machines and multilayer perceptrons. Artificial neural networks, support vector regression and regression trees are typical data mining methods for performing numeric predictions. The standard approach to select the most appropriate method for a given problem, such as a classification problem, is to perform repeated trials with multiple classifier algorithms and select the approach that performs the best on the learning problem.

More formally, prediction algorithms are typically induced through a process of supervised learning. The objective is, thus, to make predictions *y* about instances *x* of the target problem. For this, a parametrized function $\mathcal {F}: x \rightarrow y$ is induced. The prediction problem can be one of discrete value prediction, such as classifying breast cancer, or continuous value prediction, much like regression.

In order to perform supervised learning, a dataset *X* of examples, such as patient information, and corresponding values (or labels) *Y*, are compiled and used for model induction. Each row of *X* is a feature vector **x**=(*x*_1_,*x*_2_,...,*x*_*n*_). The features *x*_*i*_ are equivalent to the data variables in a statistical context. The label set, *Y*∈{*y*_1_,*y*_2_,...*y*_*n*_}, specifies the value that each corresponding instance *x*_*i*_ takes. In discrete prediction tasks, the class labels are typically mutually exclusive but do not necessarily have to be [7]. For continuous values prediction, the value space typically involves real numbers, $Y \in \mathcal {R}$, but can also apply to integers, $Y \in \mathcal {I}$.

Decision trees, Bayesian methods, support vector machines and artificial neural networks are the most common supervised learning algorithms. We provide a brief overview and direct the reader to [8] for a detailed description of these algorithms.

Decision trees are simple, but an often effective form of learning classifiers, regressors, and rules. The induction process applies a divide-and-conquer strategy which partitions the data space based on the feature values. Decision trees are often preferred over the more sophisticated models that we discuss below in fields such as medicine because the decisions leading to their predictions can be understood by humans. A very simple example of the interoperability comes from a hypothetical flu classifier which makes predictions {*FEVER*=*TRUE*∧*HEADACHE*=*TRUE*∧*COUGH*=*TRUE*→*FLU*=*TRUE*}.

The standard tree induction algorithms are CART, ID3 and C4.5 [9–11]. Decision trees are induced in a top-down manner by recursively selecting a feature that best divides the training instances according to their labels. A notion of purity known as information that is measured in units of bits is commonly used to measure purity in the determination of the best feature *f*_*i*_ at the current level *l*_*i*_. Branches from level *l*_*i*_ to level *l*_*i*−1_ are then created; one branch is made for each potential value of *f*_*i*_. The training set is partitioned based on the branches from *l*_*i*_ to *l*_*i*−1_ and the process is repeated for each node in level *l*_*i*−1_. The recursive process stops when the leaves only contain instances from a single class. It should be noted, however, that a form of pruning must be applied to the tree to avoid overfitting.

Artificial neural networks are a powerful form of learning algorithm with a long tradition in pattern recognition and machine learning. Their foundation comes from mathematical attempts at replicating information processing in biological systems [12]. In modern applications, however, they deviate significantly from the roots of their biological inspiration.

With modern memory and processing power, there is a great potential for complex artificial neural network architectures such as convolutional networks and recurrent network that have seen recent success in deep learning [2]. The standard architecture, however, is a feedforward network known as a multilayer perceptron. The name refers to the fact that the network is a directed graph that is typically composed of three or more layers. The nodes in the first layer are connected to the nodes in the second layer and so on. The first layer is the input layer. This is where the feature vector **x** enters the network. It is passed successively through the layers of the network until it reaches the final layer, the output layer. The layers between the input and the output layers are known as hidden layers. Each hidden layer is composed of a user-specified number of hidden units (the nodes in the directed graph).

For each unit *i* of each hidden layer *l*, the value of the unit $h^{(l)}_{i}$ is calculated as the values of the units connected to $h^{(l)}_{i}$ from the layer below as:

1 $$ \mathbf{h}^{(l)}_{j} = \Sigma^{d}_{i=1} x_{i} \omega_{{ji}} + b_{l},  $$

where *i* is the number of units in the previous layer, *j* is the specifies the unit in the current layer, *ω*_*ji*_ is the parametrized weights connecting layer *l*−1 to the current layer, *l*, for unit *j*, and *b*_*l*_ is the bias applied to the current layer.

An activation function is applied to hidden value $\mathbf {h}^{(l)}_{j}$. The choice of a non-linear activation, such as *sigmoid*, enables the model to learn a non-linear representation of the data. However, regularized linear units have recently been found to be useful in the hidden layers [13].

Multilayer perceptrons are typically trained via back-propagation with gradient descent. This involves updating the weights of the network over multiple iterations of the training set. This is a non-convex optimization process, and thus, training may get stuck in local minima. In practice, however, the models have been found to be very effective.

Support vector machines (SVM) are a powerful method for solving classification and regression problems based on the calculation of the maximum margin hyperplane [14, 15]. For non-linear SVM, the data is mapped to a higher dimensional space via a user-specified kernel, such as a polynomial kernel or a radial basis function. The maximal margin hyperplane is implicitly found in this higher dimensional space, the result of which can be a non-linear decision boundary in the original space. A key property of SVM is that model induction is a convex optimization problem. As a result, any local minima is also a global minima.

The maximum margin classifier is of the form *y*(**x**)=**w** ^*T*^ *θ*(**x**)+*b*, where **x** is a query instance, **w** is the maximum margin hyperplane, *θ* is a kernel function, and *b* is an offset.

The maximum margin hyperplane is solved via:

2 $$ \underset{\mathbf{w},b}{\arg \max} ~ \left\{\frac{1}{||\mathbf{w}||} ~\text{max}\left[y_{n}\left(\mathbf{w}^{T} \theta(\mathbf{x}_{n}) + b\right) \right]\right\},  $$

where **x**_*n*_ and *y*_*n*_ are the training instances and labels. Directly solving this optimization problem is very complex, however, it can be converted to a simpler, but equivalent problem using the Lagrangian dual which is solvable via quadratic programming. Finally, for kernels satisfying the property *k*(**x**_*i*_,**x**_*j*_)=*θ*(**x**_*i*_)·*θ*(**x**_*j*_) the kernel trick is used to avoid performing the computations in the kernel-space.

### Knowledge discovery

Clustering algorithms are a form of knowledge discovery performed via unsupervised learning. They group the instances of a dataset *X* into *k* clusters based on an algorithm specific notion of similarity. The process is termed unsupervised because the algorithms do not use a label set for learning. As a result, the process is one of knowledge discovery that infers the groupings from the data.

Similar to classification and regression, a wide variety of clustering algorithms have been developed. Selecting the right algorithm is domain dependent. Nonetheless, the k-means algorithm remains one of the most prominent clustering techniques. It is often preferred for its simplicity and theoretical foundation.

K-means employs an iterative process of updating the cluster centres that repeats until convergence. The *k* in k-means refers to the user-specified number of clusters. Initially, the *k* centres are set at random. Subsequently, each instance in *X* is assigned to the cluster of its nearest centre. The *k* centres are then updated to be at the centre of their assigned group. Convergence occurs when the centres stop moving.

In spite of its popularity, k-means has some well-known weaknesses, such as susceptibility to outliers. The Density-based clustering algorithm DBSCAN is an alternative method designed to account for noisy instances and outliers. In addition, one can manufacture scenarios in which k-means will fail to define good clusters under certain conditions.

Hierarchical clustering is a form of distance-based clustering that creates hierarchies of clusters. The clusters are either built agglomeratively or divisively. The former commences by assuming each instance of *X* belongs to its own cluster and builds up the hierarchy by successively merging clusters. Alternatively, the divisive approach starts with all instances in one big cluster and recursively splits the clusters into smaller clusters down the tree. This form of clustering is very effective for visualizing the groupings and different levels of granularity.

***Association rules*** are similar to the rules extracted from decision trees and produced by rule-based classifiers. The key difference is that in association rule mining, the notion of class categories is not utilized in the rule induction process.

In association rule mining, a dataset *X* is given in which the rows are instances and the columns are the feature, *F*∈{*f*_1_,*f*_2_,...,*f*_*n*_}, that quantify the instances. In medical domains, the features could be *has*_*cough*∈{*yes*,*no*}, *fever*_*level*∈{*none, low, medium, high*}, *has*_*headache*∈{*yes, no*}, *etc*.

Through association rule mining we aim to generate a set of interesting rules from *X* of the form *A*→*B*, where *A*⊂*F* and *B*⊆*F*. In contrast, rule-based classifiers learn rules of the form *A*→*B*, where *A*⊂*F* and *B*∈*Y*; here, *Y* is the set of possible class labels.

Given the definition of an association rule, any unique combination of the features, *F*, can appear on the left side and the right side of the implication. As a result, an enormous number of rules can be generated. Many of these, perhaps the majority, would be uninteresting according to any reasonable assessment. Thus, the rules must be filtered or pruned, as to only keep the valuable rules. Individually assessing each rule in a brute-force manner is prohibitive, and thus, more efficient methods of rule induction have been developed.

The APRIORI algorithm is the most common technique of association mining [16]. The key to their strategy is the employment of an iterative process that builds up frequent item sets and association rules from their simplest form (one-item sets) to the complex (two-item sets, three-item sets,..., n-item sets). An example of a one-item set and a two-item set from our medical domain is *has*_*cough*=*yes*, and *has*_*cough*=*yes*∧*fever*_*level*=*none*. The items are deemed to be frequent if they have more than a user-specified number of necessary occurrences *s* in the dataset.

The algorithm gains its efficiency from the realization that if a one-item set, such as *has*_*cough*=*yes*, is not frequent in the dataset, then no two-item set including the one-item set, such as *has*_*cough*=*yes*∧*fever*_*level*=*none*, can be frequent. Therefore, the algorithm can ignore all higher-order rules involving *has*_*cough*=*yes*. In general, the algorithm commences by finding all frequent one-itemsets and then finds candidate two-items sets from the frequent one-item sets. The two-itemsets that are frequent are kept, and the process repeats until some point, *k*, is reached where no *k*-itemsets are frequent.

In the last stage, all of the frequent itemsets are used to form association rules. The frequent item set *A*_1_∧*A*_2_∧*A*_3_, for example, generates *A*_1_→*A*_2_∧*A*_3_, *A*_1_∧*A*_2_→*A*_3_, *etc*. A similar bottom-up methodology is applied here to efficiently generate rules that meet the minimum frequency requirement.

## Methods

We have undertaken this survey in a systematic manner guided by the work of Kitchenham in [17] and the PRISMA standards [18]. Accordingly, the strategy for conducting this survey is detailed in the following sub-sections. In addition, we have taken motivation for the organization of this survey from a related survey on dengue disease surveillance [19].

### Research questions

The primary research questions considered in this survey are:

- **R1** To what degree has data mining been applied in air pollution epidemiology?
- **R2** Are there any hotbeds of this research area?
- **R3** To which sub-fields of air pollution epidemiology has data mining been applied?
- **R4** Which data mining methods have been applied?
- **R5** What are the limitations of the current work?
- **R6** What potentially fruitful directions remain unexplored?

With respect to R1, we searched the relevant epidemiological literature for research employing data mining techniques. We did not place any bounds on the dates, however, it is clear that the active period is relatively small. Moreover, there is an upward trend in the frequency as the benefits of data mining become more widely known, and tools that lower the barriers to use are made available.

Following from R1, R2 considered if the existing research is uniformly spread around the countries and institutions of the world, or if particular countries and institutions have a more keen focus on researching this area.

To address R3, we filtered through the identified articles to find any reasonable sub-categorization of the epidemiological work in terms of the application areas. This process revealed three categories of epidemiological studies of air pollution in the literature involving data mining.

In R4, we looked to see which paradigms, and which algorithms, have been applied in the air pollution epidemiology literature. From this vantage point, we found that four classes of methods have been applied.

For research question R5, we considered if, given the objectives, the data and/or the mining algorithms applied had any limitations. Given our backgrounds in data mining, we were particularly focused on the data used, algorithms applied and the processes by which the methods were evaluated.

Finally, in R6 we considered the reasonable next steps. Once again, our consideration here took a data mining perspective. To this end, we were interested in identifying new ways of using the existing data and cutting edge data mining algorithms that should be tested within this research domain.

### Search process

We performed a temporally unbounded search for articles listed in the PubMed database^1^, the Public Library of Science (PLOS)^2^ and Google Scholar^3^. This includes articles published up to the time of writing in October 2017.

The articles reported herein result from a three-part search procedure. This involved: *a*) a query-based search to produce a long list of potential articles designed and conducted by CB and MSMJ, *b*) a fine-grained manual evaluation of the long-listed articles by one author performed by CB and MSMJ, and *c*) identified articles were reviewed by the remaining authors (AOV and OZ). The queries applied to the database and with the number of articles returned are reported in Table 1.

**Table 1:** The following queries were applied to the databases

Query	Results
(“data mining”) AND ((Environment AND health) OR (exposure))	252
(“data mining”) AND (“air pollution”)	10
(“geo-spatial”) AND ((“air pollution”))	3
(“clustering”) AND (“air pollution”)	119
(“machine learning”) AND (“air pollution”)	16
(“association mining”) AND (“air pollution”)	0

We excluded articles that did not go through a peer review process in recognized biomedical publication, and articles that did not apply one or more data mining algorithms. Many environmental health articles, for example, mention, and/or discuss, the potential for data mining but did not applying data mining methods. Articles that discuss data mining in the future work were returned by our queries, but are not appropriate for inclusion in our survey.

### Data extraction and synthesis

The following information was extracted from each of the selected articles:

- The source (journal or conference) and full reference.
- A summary of the objective of the study.
- The air pollutants of interest in the study.
- The data mining method applied to achieve the objective.
- A summary of the findings of the study.

This information was extracted by CB and MSMJ and validated by AOV and OZ. Any disagreements were handled via discussion and common consensus. After the raw details of the articles were tabulated, data synthesis was performed. In addition, AOV extracted information about each article related to the biomedical objectives.

Data synthesis involved analyzing the objectives, data mining methods, and the target pollutants in order to identify categories to effectively group the various studies. This exercise was performed by CB and reviewed by the remaining authors. Our goal in the categorization was to identify a hierarchy of categories that provided a sketch of the research landscape. In addition, the purpose was to facilitate quick and easy locating of the studies that are related to the reader’s area of interest. The identified categories are listed below:

- Physical Area
  - Indoor
  - Outdoor (Rural, Urban and General^4^)
  - General
- Objective
  - Forecasting and Prediction
  - Source Apportionment
  - Hypothesis Generation
- Data Mining Method
  - Regression
  - Classification
  - Clustering
  - Association Mining

## Aspects of data mining in air pollution epidemiology

### Environmental setting: overview

In this section we discuss the target areas of interest (the environmental setting). We have separated these into indoor, outdoor and general. Indoor refers the studies focused on indoor air pollution, such as air pollutants measured within the home or workplace. Outdoor refers to studies interested in outdoor air pollution, such as air pollution measured at a specific intersection or the dispersion of pollutants across an area of interest. It can be further separated into urban, metropolitan and rural. Given that the current breadth of research is still relatively sparse, we focus on the top level of abstraction in this article. We note, however, that a large portion of the research in the outdoor category has been applied to urban and/or metropolitan settings. This is, perhaps, not surprising given that the high population density in metropolitan areas can lead to high impact research. Nonetheless, it suggests rural environments as a potential direction for future work.

The general category covers research that applies data mining methods to study the health impacts of combinations of chemicals common in air pollution. These studies were typically conducted in laboratory settings rather than in the field (or relying on data collected from the field). Table 2 includes a categorized list of articles in relation to their environmental settings.

**Table 2:** Categorization of articles organized by the application setting

Setting	References	*n*(*%*)
Outdoor	[4, 20, 22–24, 26, 27, 30–36, 38, 39, 43–60]	87
Indoor	[55, 61, 62]	8
General	[63, 64]	5

The final column (*n*(*%*)) is the percentage of articles in each category

### Categorized study objectives: overview

We grouped the selected articles into the following general study objectives: forecasting and prediction, source apportionment and hypothesis generation. A large percentage of the articles identified in our survey dealt with forecasting or predicting pollution levels based on various climatic and/or pollutant values. These studies considered: *a*) forecasting future pollution levels at a specific location given some specific data for that location, *b*) forecasting current pollution levels at a specific site given some regional data, and *c*) forecasting the geo-spatial distribution of air quality or the spread of pollutants.

Closely related are the studies that were designed to predict increases in sickness or hospitalization from climatic and pollution measurements or to classify sickness in individuals given an air quality or pollution assessment.

Studies classified into the source apportionment category aimed to trace a given decrease in air quality or increase in a given pollutant back to its emission source given a set of pollutant and climatic variables.

Finally, a large number of articles performed hypothesis generation. These studies take in the wide variety of data available about the evolution of air pollution at a specific location, its spread across a region or the globe, health indicator variables, *etc*., and use data mining algorithms to identify hidden associations between the variables. These associations are used to test existing assumptions and generate new ones. Exemplary associations might indicate that a certain chemical combination *X,Y,Z* is associated with increased volume in the emergency department at a hospital of interest, or that climatic conditions *W,R* combined with heavy seaport traffic, lead to a decrease in the air quality index. These associations can serve to motivate focused trials to study the discovered relationship in depth.

Table 3 includes the articles in a list sorted according to the objectives of the research. It is worth noting that a given article may have more than one objective, and thus, may appear multiple times in the table.

**Table 3:** Categorization of articles organized by the study objective

Setting	References	*n*(*%*)
Forecasting	[4, 24, 26, 27, 30, 33–35, 38, 39, 43, 44, 47–49, 52, 54–62, 65–69]	60
Source apportionment	[22–24, 45, 51]	10
Hypothesis generation	[20, 22, 31, 32, 35, 36, 38, 45, 46, 48, 50, 51, 53, 64, 69]	30

The final column (*n*(*%*)) is the percentage of articles in each category

## Results

### Summary statistics

#### PRISMA results

**Fig. 1:**
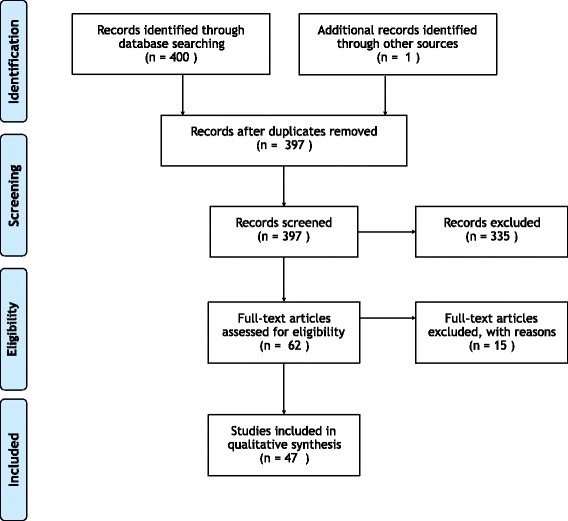
PRISMA flow diagram. Overview of the PRISMA results from our search process

The summary statistics recording the numbers of articles returned from our search process, excluded, and included are presented in the PRISMA flow chart in Fig. 1. Our initial search returned 400 articles. In addition to these, one article ([20]) was suggested during the review process. After the initial screening and eligibility assessment, 47 articles were included in this survey.

#### Regional and temporal overview

**Fig. 2:**
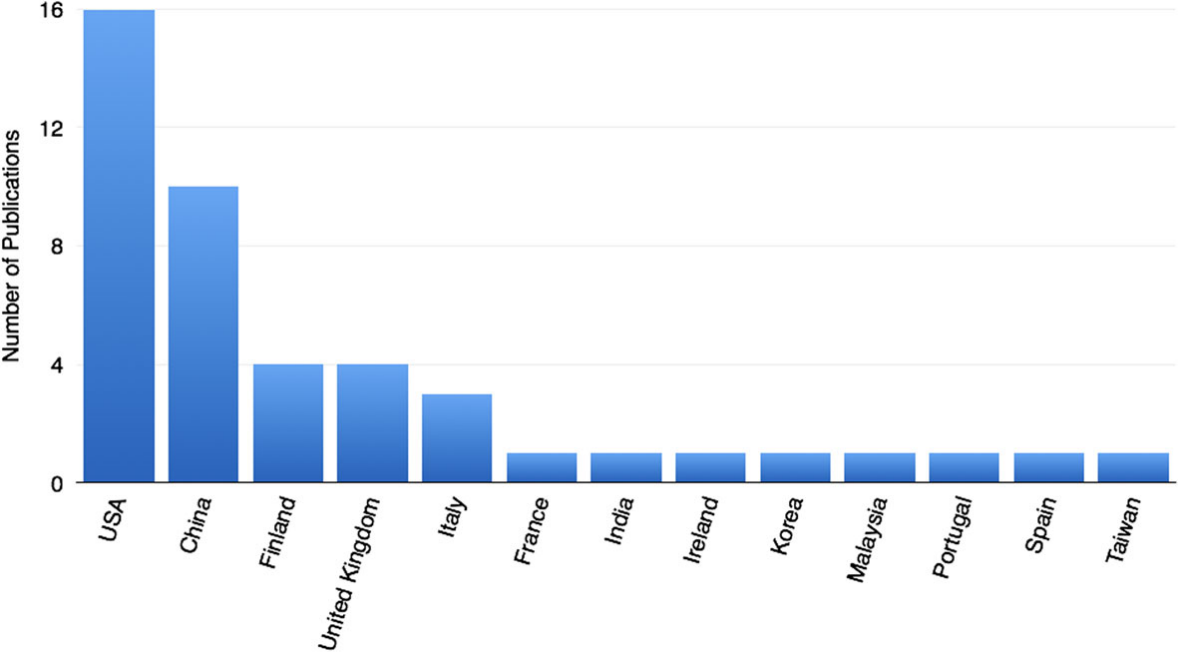
Publications Per Country. The number of publications per country identified a predominance in the filed by European countries, the USA and China

**Fig. 3:**
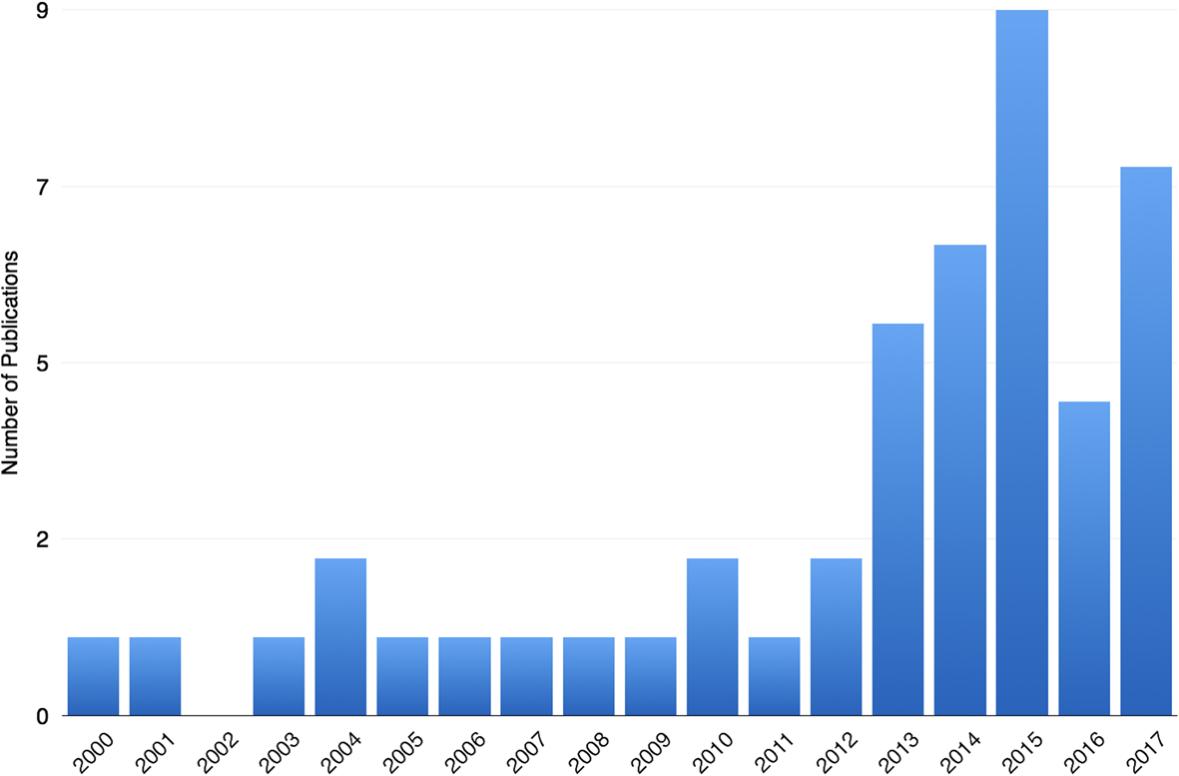
Publications Per Year. Number of articles per year between January 2000 and October 2017. We identified an apparent tendency of an increased number of publications on data mining and epidemiology in recent years

We have found that eighteen of the studies were from Europe and the UK, sixteen were from the USA, ten were from China, and four were from other Asian countries. The detailed breakdown of this is provided in Fig. 2. The papers were published between 2000 and October 20, 2017. Figure 3 illustrates a strong upward trend in recent years. We believe this to be owing to better access to data and computing power, along with a growing awareness and access to data mining tools that are accessible to users outside of the data mining community. These tools include the Weka data mining software, which enables users to directly apply data mining algorithms to their data through Java interfaces or a graphical user interface [21].

#### Study objectives

The summary statistics for the study objectives are as follows: sixty percent of the study objectives were to forecast or predict epidemiological values/outcomes, such as the AQI or increases in emergency room visits. Thirty percent performed hypothesis generation. This included objectives, such as learning from the data, in which combinations of variables are associated with increases in hospitalization, and understanding which combination of meteorological variables are associated with a degradation in air quality due to emissions from neighbouring cities. Finally, ten percent of the studies focused on source apportionment.

#### Data mining paradigm

We identified that classification, regression, clustering and association mining algorithms have been applied. Classification and regression relate to prediction and forecasting objective, whereas clustering and association mining generally apply to hypothesis generation and source apportionment.

Table 4 includes the articles in a list sorted according to the objectives of the research. Data mining methods for performing numeric predictions, such as regression and classification, were most widely applied. This area encompassed 59% of the research. Clustering algorithms were applied in 26% of the work, and 15% of the articles employed association mining.

**Table 4:** Categorization of articles organized by the data mining approach

Setting	References	*n*(*%*)
Prediction	[4, 20, 24, 24, 26, 27, 30, 31, 33, 34, 47, 49, 50, 52, 54, 59–62, 65–68]	59
Clustering	[22, 23, 43–46, 51, 55, 58, 69]	26
Association Mining	[32, 35, 36, 48, 53, 63]	15

The final column (*n*(*%*)) is the percentage of articles in each category

### Detailed analysis

#### Source apportionment

Table 5 summarizes source apportionment studies employing data mining techniques. These studies explore the impact of chemical emissions and other airborne agents in conjunction with climatological factors [22–24]. They focus on apportioning particular airborne pollutants to potential sources, such as industrial sites, regions and major intersections. These studies have mainly focused on outdoor and urban air pollution as it is the most widely known issue. In particular, principal component analysis (PCA) has been applied to identify correlations and the importance of particular meteorological parameters, traffic, fuel fired equipment and industries in causing air pollution [23–25]. Alternative approaches have utilized clustering-based solutions with correlation analysis to accomplish the task of source apportionment [22, 23].

**Table 5:** Summary of air pollution source apportionment studies using data mining techniques

Author	Year	Sub-field	Environmental agent of interest	Data mining techniques	Objective
Chen et al. [22]	2010	Outdoor air pollution	Inorganic acids & basic air pollutants	Hierarchical Clustering	Explore relationship between climate and air pollutants
Singh et al. [24]	2013	Outdoor air pollution	AQI	PCA, SVM, DT	Predicting air quality and identifying air pollution sources.
Fernández- Camacho et al. [51]	2015	Urban air and noise pollution by traffic	NOx, O3, SO2, Black Carbon	Fuzzy Clustering	Find the relationship of noise to the traffic emission
Chen et al. [23]	2015	Outdoor air pollution	Multiple air pollutants	Clustering	Source apportionment for air pollutants
Li et al. [45]	2017	Outdoor air pollution	PM	Trajectory clustering	Use clustering to understand how seasonality and meteorology effects pollution sources for Beijing

Chemical abbreviations: *AQI* air quality index, *NOx* nitrogen oxides, *O3* ozone, *SO2* sulfur dioxide, *PM* particulate matter. Data mining abbreviations: *PCA* principle component analysis, *SVM* support vector machine and *DT* decision tree

***Strengths***: The work presented in [23] proposes to perform enhanced source apportionment and classification. The authors claim that the key to achieving this is in the use of clustering algorithms developed for data mining. The advantage of these is that they are intended for rich, high-dimensional datasets that may include outliers. These factors can be problematic for conventional methods of source apportionment, such as principle component analysis and positive matrix factorization.

Once again, we have identified that the clarity with which the authors present the problem, and then juxtapose the limitation of conventional methods with the potential of data mining approaches, to be a very strong point in this paper. In addition, we appreciate that the authors have gone beyond simply applying standard clustering algorithms, and rather, employed their domain knowledge in order to refine the method in order to develop a superior clustering algorithm for the domain. The authors describe their algorithm, how to set the threshold parameter and the data pre-processing in detail. Crucially, this makes the proposed solution easily implementable by others.

#### Forecasting and prediction

Tables 5 and 6 summarizes 18 studies which applied machine learning techniques. We observed that investigators are primarily interested in predicting a) the distribution of ambient pollutant concentrations or related measures such as the air quality index (AQI), b) human exposure or c) risk of a health outcome.

**Table 6:** Summary of studies forecasting air pollution distributions and related variables using data mining methods

Author	Year	Sub-field	Environmental agent	Data mining techniques	Objective
Kolehmainen et al. [60]	2001	Outdoor air pollution	NO2	ANN	Comparing two Neural Nets for their suitability in forecasting Air Quality
Kukkonen et al. [33]	2003	Outdoor air pollution	PM NO_2_,	ANN	Machine Learning Model comparison for forecasting NO2 and PM10 concentrations
Niska et al. [59]	2004	Outdoor air pollution	NO2	Genetic Algorithms, ANN	Investigate the use of GA to find a better ANN model to forecast air quality
Ghanem et al. [69]	2004	Outdoor air pollution	SO2,C6H6,NO,NO2,O3	Hierarchical clustering	Monitor chemicals and outline challenges related to collection and processing.
Corani [68]	2005	Outdoor air pollution	Ozone, PM10	ANN, Lazy Learning	Predict levels of air pollutants from meteorological and other local variables.
Dominici et al. [67]	2006	Outdoor air pollution	PM2.5	Bayesian Hierarchical Models	Assess the association of air pollution levels with the number of deaths per day
Ma et al. [58]	2008	Outdoor air pollution	SO2, O3, NOx, C6H6	k-means	Developing a distributed air pollution monitoring system & use data mining to find patterns of pollutant distribution
Pegoretti et al. [62]	2009	Indoor air pollution	Rn	Geostatistical Models, KNN	Forecasting the indoor Radon concentrations
Aquilina et al. [39]	2010	Outdoor air pollution	particle-associated PAH	DT, ANN	Predict personal exposure to particle-associated polycyclic aromatic hydrocarbons (PAH)
Padula et al. [57]	2012	Outdoor air pollution	Traffic-related pollution	Targeted maximum likelihood estimation	Estimate the probability of low birth weight among full-term infants based on the mother’s exposure to traffic-related air pollution
Zhu et al. [35]	2012	Urban outdoor air pollution	SO2, NO2, PM10, Respiratory diseases	ARM, GMDH	Forecasting the number of respiratory patients based on the seasonal effects of air pollution
Singh et al. [24]	2013	Outdoor air pollution	AQI	PCA, Ensemble Decision DT, SVM	Predicting the Air Quality and identifying major sources of air pollution
Beckerman et al. [66]	2013	Outdoor air pollution	NO2, PM2.5	GLM	Develop a better land use regression model for using machine learning methods
Pandy et al. [38]	2013	Outdoor air pollution	UFP, PM	DT, RF, *etc.*	Test machine learning classifiers for predicting air quality and assess the impact of weather and traffic related variables on UFP and PM.
Philibert et al. [56]	2013	Setting	*N*_2_0	RF	Predict NO2 emissions using variables related to chemical fertilizer treatments applied to agricultural plots.
Chen et al. [54]	2014	Outdoor air pollution	Smog	ANN, Social Network Analysis	Predicting Smog based Health Hazardous regions
Dias et al. [55]	2014	Outdoor air pollution	PM2.5	Density-based Clustering	Quantification of human exposure to traffic related air pollution
Lary et al. [4]	2014	Outdoor air pollution	PM2.5	Ensemble Algorithms RF, SVM, ANN	Estimating the daily distributions of PM2.5
Jiang et al. [26]	2015	Outdoor air quality	AQI	Correlation Analysis	Monitoring the dynamics of air quality in large cities based on social media
Wang et al. [27]	2015	Outdoor air pollution	Generic	Topic Models LDA, NLP	Evaluating the use of social media data to estimate air pollution and public response
Reid et al. [34]	2015	Outdoor air pollution	PM2.5	Generalized boosting model, GAM, RF, SVM, KNN Regression, etc.	Predicting PM2.5 during wildfire
Lary et al. [52]	2015	Outdoor air pollution	PM2.5	Ensemble regression models	Estimating PM2.5 distribution and relationship of such air pollutants with mental health
Lewis et al. [49]	2016	Outdoor air quality	NOx,O3, SO2, CO, VOCs, PM	Boosted regression DT, gaussian process emulation	Improve the accuracy of common low cost air pollution sensors
Hu et al. [65]	2016	In/Outdoor air pollution	Generic	RF	Understanding, exposure to air pollution by predicting time-activity tracking of individuals
Challoner et al. [61]	2015	Indoor air pollution	PM NO2,	ANN	Predicting the indoor air quality from outdoor monitors
Mirto et al. [48]	2016	Outdoor air pollution and climate	Generic	Spatial data mining, hot spot analysis	Finding correlations between diseases and air pollution due to climatic factors
Xu et al. [30]	2017	Outdoor air pollution	PM, CO O3, SO2 NO2,	SVM, Fuzzy Evaluation, Empirical Mode Decomposition	Air quality forecasting and evaluation
Min et al. [43]	2017	Outdoor air pollution	PM2.5	K-Means	Apply K-Means to the identify potential new monitoring sites by considering a larger set of 313 variables in their models. Traffic and urbanicity are found to be useful to guide site selection
Keller et al. [44]	2017	Outdoor air pollution	PM2.5	Modified K-Means	A clustering method to assess exposure to air pollution in health-related studies. They consider the multivariate nature of the exposure and spatial misalignment likely to occur when using data from central monitoring stations and the actual location of the cases
Liu et al. [47]	2017	Outdoor air pollution	PM, SO2, CO, NO2, O3	SVM Regression	Apply support vector regression for air pollution forecasting using six criteria pollutants, five meteorological conditions and the Air Quality Index

Chemical abbreviations: *NO* nitrogen oxide, *NO2* nitrogen dioxide, *NOx* nitrogen oxides, *UPM* ultra fine particulate matter, *PM* particulate matter, *SO2* sulfur dioxide, *C6H6* benzene, *O3* ozone, *Rn* radon, *AQI* air quality index, *VOCs* volatile organic compounds. Data mining abbreviations: *ANN* artificial neural network, *DT* decision trees, *ARM* association rule mining, *GMDH* group method of data handling, *PCA* principle component analysis, *SVM* support vector machine, *GLM* generalized linear model, *RF* random forest, *LDA* latent dirichlet allocation, *NLP* natural language processing, *GAM* general additive models, k-nearest neighbors. Note, k is a constant value specifying the number of nearest neighbors in kNN and the number of clusters in k-means

According to the above points, the first category consists of 15 studies which either focus on predicting the distribution of particular air pollutants or predicting the quality of air in general. Twenty-seven percent of the above 15 studies (i.e. 4) focus on predicting or forecasting the air quality or air pollution in general. Sixty-six percent of the studies (i.e. 10) are interested in well-known specific air pollutants such as nitrogen oxides (NOx), particulate matter (PM), sulfur dioxide (SO2), carbon monoxide (CO), ozone (O3) and Volatile Organic Compounds VOCs. An interesting, and potentially fruitful, data source utilized by some of the studies that focus on air quality prediction comes from social media posts; social media offers a very rich source of information, which is not typically utilized in scientific analyses of this type [26, 27].

Fifty percent of the studies focusing on specific air pollutants use artificial neural networks. Other well-known data mining techniques used include decision trees and support vector machines. In addition, some studies have used ensemble models which are composed of multiple models. The final outcome is determined based on the consensus of the outcome of each model, or some other method of arbitration. Ensemble models have been demonstrated to outperform the base classifiers from which they are composed in a variety of settings. They can be applied to both discrete and continuous value prediction [28, 29].

The second category, namely human exposure, consists of 4 studies. These studies focus on identifying regions or exposures, predicting the activities of humans to help understand the exposure better and quantifying the exposure levels.

Interestingly, some of the studies also focus on building better infrastructure to collect data, or on ways to improve the quality of the collected data. This investment can be interpreted as a level of confidence in the application of data mining, and its potential to help shape future research and understanding. The interest in more fundamental problems like data collection and the accuracy of the collected data, in addition to a single focus on building a model based on the available data is very important. Work based on primary questions as such these will ensure high-quality datasets are available in the future, and thus, that better data mining and machine learning models will be possible.

***Strengths***: The authors in [30] propose a hybrid system that incorporates a variety of machine learning methods to produce more accurate forecasts and evaluations of air pollution. The authors note that data driven approaches are often more accurate and less complex than model-based approaches, such as chemical transport models, for predicting air quality. Data mining and machine learning-based approaches are data driven methods that are recognized as being powerful forecasting tools. This motivates them as a good choice for the authors. Although we do not see this as being as strong as the previous motivations for applying data mining and machine learning, it is certainly a sufficient reason to consider machine learning.

#### Hypothesis generation

We observed that many studies (i.e. above 60% of the studies that we have considered) have predominantly applied association rule mining—a primary class of data mining techniques—to generate new hypotheses regarding potential connections between air pollution and adverse health conditions.

From the identified articles, we observed that respiratory disease is an adverse health outcome of interest in these studies. Many studies focusing on respiratory disease are interested in finding out any potential connection between the disease and particulate matter or other airborne pollutants such as SO2 and NOx.

Our results demonstrated that there is a growing interest in generating new hypotheses explaining the connection between a combination of air pollutants and a particular adverse health impact. In [31], for exampled, the authors used the Bayesian Kernel Machine Regression (BKMR) method, which was recently introduced by epidemiologists. This illustrates the benefit of applying data mining methods to modern epidemiological datasets.

***Strengths***: The authors in [20] are interested in generating hypotheses about the joint effect of multiple airborne chemicals on pediatric asthma. Their work demonstrates that classification and regression trees can be used to overcome the challenge presented by multiple chemical interactions when identifying complex joint effects.

We have identified this as a noteworthy paper because the authors are studying a problem that is difficult to solve using conventional epidemiological methods. The paper is strengthened by the fact that the authors clearly justify the ML/DM solution to the problem. In addition, the authors explain why they selected the specific ML algorithm. Finally, this work is an excellent example of how ML/DM algorithms can be augmented and combined with knowledge and practices from the target domain in order to make an accurate and appropriate joint methodology. In particular, the authors demonstrate a refinement to the standard CART algorithm to control for confounding variables. This is important to note because in some applications, data mining practitioners can lose sight of useful, and often necessary, domain knowledge, which hampers the final results. Table 7 summarizes 15 studies which applied data mining techniques to generate new hypotheses to better understand the relationship between air pollution and health.

**Table 7:** Summary of hypothesis generating studies using data mining methods to generate new hypotheses to understand the relationship between air pollution and health conditions better

Author	Year	Sub-field	Environmental agents	Data mining techniques	Objective
Chen et al. [22]	2010	Outdoor air pollution	Inorganic acids & basic air pollutants	Hierarchical Clustering	Explore relationship between climate and air pollutants
Zhu et al. [35]	2012	Urban outdoor air pollution	SO2, NO2, PM10, Respiratory diseases	ARM, GMDH	Forecasting the number of respiratory patients based on the seasonal effects of air pollution
Pandy et al. [38]	2013	Outdoor air pollution	UFP, PM	DT, RF	Test machine learning classifiers for predicting air quality and assess the impact of weather and traffic related variables on UFP and PM.
Payus et al. [32]	2013	Outdoor air pollution	SO2, NO2, PM10, CO,O3	ARM	Find associations between combinations of air pollutants with respiratory illness.
Bobb et al. [31]	2014	Mixture of chemicals	Multiple chemicals, neurodevelopment, hemodynamics	Bayesian kernel machine regression (BKMR)	Identifying mixtures (e.g., metals) and components responsible for various health effects (e.g., neurodevelopment)
Gass et al. [20]	2014	Outdoor air pollution	CO, NO2, O3, PM	Classification and regression trees	Apply classification and regression trees to generate hypothesis about exposure to mixtures of pollutants and health effects. They work with children’s asthma emergency visit
Fernández-Camacho et al. [51]	2015	Urban air and noise pollution by traffic	NOx, O3, SO2, Black Carbon	Fuzzy clustering	Find the relationship of noise to the traffic emission
Bell et al. [63]	2015	General chemical exposure	219 chemicals	ARM	Find relationships between chemicals and health biomarkers or diseases
Qin et al. [53]	2015	Outdoor air pollution	PM	ARM	Exploring relationships of PM spatial-temporal variations and how cities influence each other
Reid et al. [50]	2016	Outdoor air quality with wildfire	PM2.5 Respiratory diseases	Generalized estimating equation and generalized boosting model	Finding the relationship between wildfire and associated increment in PM2.5 affects people with respiratory diseases
Toti et al. [36]	2016	Outdoor air pollution, pediatric asthma	SO2, NO, PM, NO2	ARM	Exploring relationships of Air Pollution Exposure on Asthma
Mirto et al. [48]	2016	Outdoor air pollution & climate changes	Generic	Spatial data mining, hot spot analysis	Finding correlations between diseases (e.g. respiratory and cardiovascular diseases, cancer, male human infertility) and air pollution due to climatic factors
Li et al. [45]	2017	Outdoor air pollution	PM	Trajectory clustering	Apply clustering to identify transport pathways, sources and seasonal variations of particulate matter (PM2.5 and PM10) in Beijing for regulation purposes
Stingone et al. [46]	2017	Outdoor air pollution	National air toxics assessment	DT	Apply machine learning to identify air pollutants exposure profiles when exploring multiple pollutants (104 ambient air toxics) and then estimate the magnitude of the profile’s effect on math scores in kindergarten children
Ghanem et al. [69]	2004	Outdoor air pollution	SO2,C6H6,NO, NO2,O3	Hierarchical clustering	Monitor chemicals and outline challenges related to collection and processing.

Chemical abbreviations: *SO2* sulfur dioxide, *NO* nitrogen oxide, *NOx* nitrogen oxides, *NO2* nitrogen dioxide, *UFP* ultra fine particulate matter, *PM* particulate matter, *O3* ozone and *C6H6* benzene. Data mining abbreviations: *ASM* association rule mining, *GMDH* group method of data handling, *DT* decision tree and *RF* random forest

## Discussion

### Challenges and limitations

We have identified a few reoccurring challenges in the surveyed papers. A major theme revolves around data. Many articles, for example, report results from data collected over a short period of time, and from one, or only a few, locations [32, 33]. As a result, the findings cannot necessarily be generalized to new locations. This is particularly the case for prediction models trained on local data.

Most real-world data requires preprocessing to combine data sources, remove noise and properly structure the data. The necessities of this may be challenging for domain practitioners. Moreover, certain decisions that must be made during preprocessing can have an impact on the effectiveness of the trained model. Decision trees and association mining algorithms, for example, take categorical variable inputs, whilst continuous variables are common in epidemiology and atmospheric science. Thus, variables, such as temperature, must be converted to discrete categories (low, medium, high) for example [33]. In many cases, the ideal split points may be unclear. In general, the current literature does not focus on how to best preprocess air pollution epidemiological datasets.

Given the volume of social media data, and the fact that the vast majority of it is irrelevant to the data mining objective, it often has to be filtered. In [27], for example, keyword filtering is applied to gather relevant micro-blogs from Sina Weibo. How exactly to filter, or process the data, is an open question. A potential new direction here is to apply feature selection or feature extraction [2].

As noted by [34], it is important to recognize the limits of your data. Issues, such as granularity and representativeness, can limit what can be discovered from the data. Likewise, when generating association rules to predict outcomes, such as hospitalization or an increase in respiratory disease from weather and pollution data, the training data may not account for all relevant factors. In [35], it is noted that their data does not account for the accumulative nature of health outcomes.

Other challenges in applying data mining methods include the selection of user-specified parameters for the algorithms. Choosing the ideal number of clusters, for example, is important for performance of clustering algorithms [22]. In addition, metrics must be used that are appropriate for the target domain. In some cases, suitable evaluation metrics may not exist within the data mining literature, in which case new metrics may be required [36].

Finally, in many cases practitioners prefer data mining models that produce predictions in a manner that can be easily analyzed and understood [32, 36]. This limits the choice of algorithms to rule learners and decision trees, and thus, many of the strongest algorithms are omitted. Perhaps, research focused on making the predictions of artificial neural networks and support vector machines more interpretable could be helpful for the health sciences community [37].

From an application perspective, we found the discussion of data mining related choices to be limited. Whilst the majority of the articles surveyed contain sufficient details about the algorithms implemented, readers could benefit from a similar level of detail in regards to other key design and implementation decisions. With few exceptions, such as [38], most of the surveyed articles report results for a single data mining algorithm. Readers would benefit from understanding how and why the specific choice of algorithm was made. As we noted in the overview section on Paradigms of Data Mining Algorithms, it is standard within the data mining community to run trials using a diverse set of algorithms. We often missed a discussion of details regarding which other algorithms were considered, and how they were evaluated. Finally, details regarding how the software was implemented and which data mining packages were used would be valuable to other readers from the air pollution epidemiology community.

### Future directions

#### Deep learning

Traditional artificial neural networks have proven to be accurate predictors for classification and regression problems. Within this survey, we have found them to be used for predicting global ground level *PM*_2.5_ [4], predicting air pollution indicators and modeling personal exposure [39]. In recent years, however, deep learning has elevated the potential for learning with artificial neural networks to new heights. Thus, deep learning methods may also be very fruitful within air pollution epidemiology.

Deep learning is based on standard artificial neural network algorithms but utilizes much larger and deeper networks trained on big datasets. The training process in conjunction with the depth of the networks enables the learning of data abstractions at the different depths. This is found to disentangle complex features. Deep learning methods have been highly effective in areas such as image classification, speech recognition, and other complex problems [2].

#### Model selection

Model selection and evaluation are very important aspects of applying machine learning algorithms to real-world applications. However, they often receive less attention than the machine learning algorithms themselves. It is important to consider the breadth of techniques when developing applications in data mining in order to select the right approach for the domain.

For a given machine learning algorithm, model selection refers to the choosing of a parameterized version of the model based on the training data. The key is to select a model that will perform well on unseen data in the future. Once a parametrized model has been selected, the evaluation process provides an estimate of how the model will perform during future application. Some common evaluation metrics are accuracy, root mean square error (RMSE), f-measure and the area under the ROC curve (AUC).

#### Cross-validation

In the surveyed literature, various forms of cross validation have been applied [24, 26, 38]. In addition to these, various other methods can be applied, each of which has strengths and weaknesses. It is important to select a method that is appropriate for your target domain. Evaluation metrics estimate performance in different ways, and thus, it is important to choose one that is consistent with the target domain. The details of model selection and evaluation are thoroughly discussed in [6].

#### Association mining

Our results demonstrated that much of the research that applied hypothesis generation utilize association mining. These studies typically relied on frequency to identify the associations. It is worth pointing out some alternatives, particularly for scientific domains. Statistical significance test-based methods, for example, have been developed to offer a better assessment of the quality of the association [40, 41]. These could be of great benefit to future applications in air pollution epidemiology.

#### Class imbalance

In a related context, [38] noted the potential impact of class imbalance, or skewed class distributions, on the performance of machine learning algorithms. Class imbalance is said to occur when one class is significantly less likely, or less frequent, in the training set, than the other class. A detailed discussion of the impacts and potential solutions to class imbalance is undertaken in [42]. Given that we are often interested in less frequent, or even rare, events in air pollution epidemiology, methods developed for imbalanced learning may have great potential here.

## Conclusion

Recent progress in technology and corresponding decreases in the price of computing power has made if possible to measure and store a wide variety of environmental health variables and form them into big datasets. Moreover, social media and other on-line resources provide an entirely new perspective from which to conduct environmental health analyses. These big datasets come with complexities that render it difficult to rely on traditional epidemiological or environmental health models to analyze them. To this end, data mining methods offer great potential to advance our understanding of the causes and impacts of air pollution.

From our survey, we have found a strong increase in the number of articles reporting to apply data mining methods to air pollution epidemiology. We attribute this to the increasing availability of large datasets and computing power, along with the growing awareness of the potential benefits of data mining. In spite of this trend and the potential benefit within the field, to the best of our knowledge, a survey of the existing state-of-the-art has not been performed.

To fill this void, we undertook a study to explore the extent to which data mining has been applied to air pollution epidemiology. This survey is intended for practitioners and researchers alike. We aim to point domain researchers to existing data mining applications within their respective areas, and related areas, as well as advance their understanding of the potential of data mining and inspire them to explore further research avenues.

Our survey illustrates that a wide variety of data mining algorithms have been applied to various sub-fields of air pollution epidemiology. Machine learning algorithms, for example, have been applied both as classifiers and regressors in forecasting and prediction problems. Clustering algorithms, such as K-Means and hierarchical clustering have been applied to knowledge discovery and source appropriation. In addition, a great number of studies have applied association mining for hypothesis generation.

## Endnotes

^1^ see https://www.ncbi.nlm.nih.gov/pubmed

^2^ https://www.plos.org

^3^ https://scholar.google.ca

^4^ General refers to the study of air pollutants not specific to a certain setting.
